# Preventive treatment with liraglutide protects against development of glucose intolerance in a rat model of Wolfram syndrome

**DOI:** 10.1038/s41598-018-28314-z

**Published:** 2018-07-05

**Authors:** Maarja Toots, Kadri Seppa, Toomas Jagomäe, Tuuliki Koppel, Maia Pallase, Indrek Heinla, Anton Terasmaa, Mario Plaas, Eero Vasar

**Affiliations:** 1 Institute of Biomedicine and Translational Medicine, Department of Physiology, University of Tartu, 19 Ravila Street, Tartu, 50411 Estonia; 2 Institute of Biomedicine and Translational Medicine, Laboratory Animal Centre, University of Tartu, 14B Ravila Street, Tartu, 50411 Estonia; 3 Centre of Excellence for Genomics and Translational Medicine, University of Tartu, Ravila 19, Tartu, 50411 Estonia

## Abstract

Wolfram syndrome (WS) is a rare autosomal recessive disorder caused by mutations in the *WFS1* (Wolframin1) gene. The syndrome first manifests as diabetes mellitus, followed by optic nerve atrophy, deafness, and neurodegeneration. The underlying mechanism is believed to be a dysregulation of endoplasmic reticulum (ER) stress response, which ultimately leads to cellular death. Treatment with glucagon-like peptide-1 (GLP-1) receptor agonists has been shown to normalize ER stress response in several *in vitro* and *in vivo* models. Early chronic intervention with the GLP-1 receptor agonist liraglutide starting before the onset of metabolic symptoms prevented the development of glucose intolerance, improved insulin and glucagon secretion control, reduced ER stress and inflammation in Langerhans islets in Wfs1 mutant rats. Thus, treatment with GLP-1 receptor agonists might be a promising strategy as a preventive treatment for human WS patients.

## Introduction

Wolfram syndrome (WS) is a rare neurodegenerative disorder caused by biallelic mutations of the Wolframin1 (*WFS1)* gene and whose main symptoms are diabetes mellitus, optic nerve atrophy, hearing loss, and neurodegeneration in the brainstem^1–3^. Unfortunately, no effective cure exists for WS, and currently treatment is focused on relieving symptoms^4^. Corrupted function of WFS1 leads to impairment of cellular calcium regulation and increase in endoplasmic reticulum stress, ultimately causing cell death. Thus, one possible strategy for treatment of WS is to normalize cellular calcium regulation and reduce endoplasmic reticulum stress^4^.

GLP-1 (glucagon-like peptide-1) receptor agonists have been accepted as a promising class of anti-diabetic drugs, having the potential to delay or even reverse disease progression^5^. An incretin peptide, GLP-1 regulates glucose homeostasis, metabolism, and neural survival directly on tissues expressing GLP-1 receptors and indirectly through neuronal and endocrine pathways^6,7^. Acute treatment with the GLP-1 agonist exenatide has shown a promising anti-diabetic effect in Wfs1 knock-out mice^8^. The GLP-1 agonist Exendin-4 was shown to reduce endoplasmic reticulum (ER) stress in cardiac myocytes and prevent a decrease in activity of sarco/endoplasmic reticulum Ca-ATPase-2a (SERCA2a)^9^. Early intervention with the GLP-1 receptor agonist liraglutide improved glucose tolerance and beta cell apoptosis in prediabetic Gato-Kakizaki rats^10^. Considering these results, treatment with GLP-1 agonists appears to be an efficient way to reduce elevated ER stress *in vitro* and *in vivo*. Thus, GLP-1 agonists could be a feasible strategy for managing WS.

**Figure 1:**
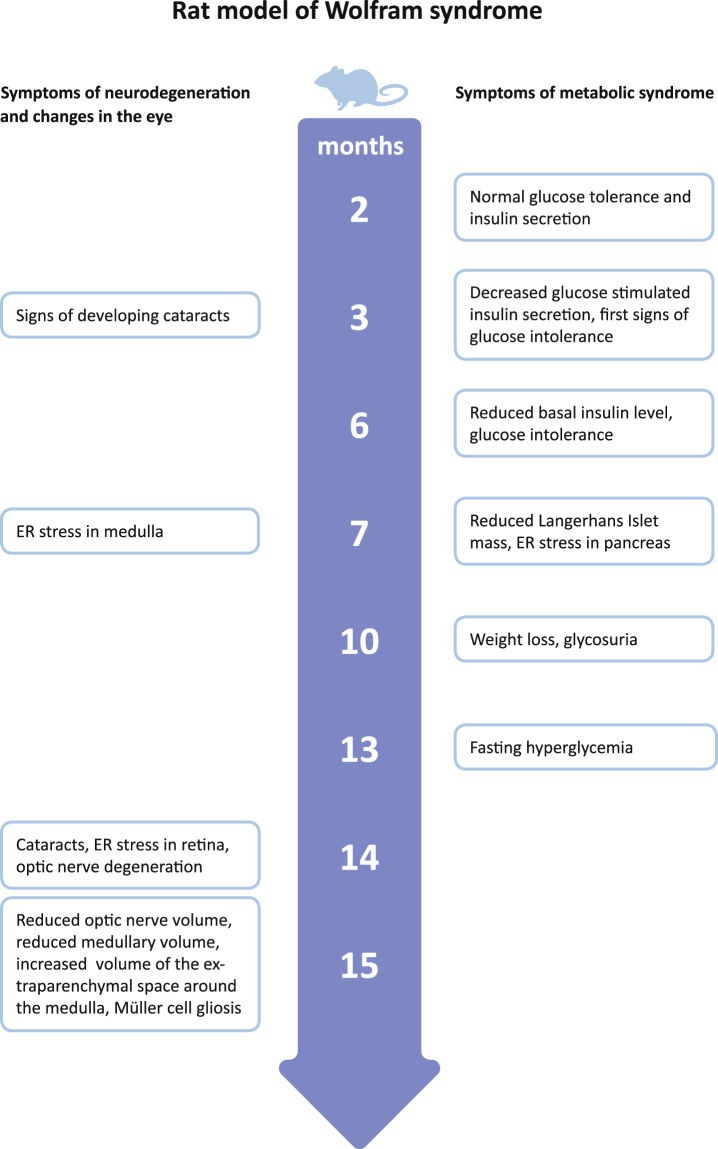
WS development in Wfs1 deficient rat. Based on Plaas *et al*.^11^.

Recently, a rat model of WS has been developed and validated^11^. Deletion of exon 5 of the *Wfs1* gene resulted in development of the main symptoms of WS: diabetes mellitus (Fig. 1), medullary degeneration, and optic nerve atrophy^11^. Moreover, levels of ER stress were elevated in the pancreas and brainstem of this rat^11^. The progressive development of diabetes mellitus and other WS symptoms makes the Wfs1-ex5-KO232 rat a good tool to study treatment options. Therefore, the aim of the present study was to investigate the effect of chronic treatment with GLP-1 receptor agonist on the progression of glucose intolerance in a Wfs1 mutant rat.

## Results

### One-week treatment with liraglutide restores glucose tolerance in 5-month-old Wfs1 KO rats

**Figure 2:**
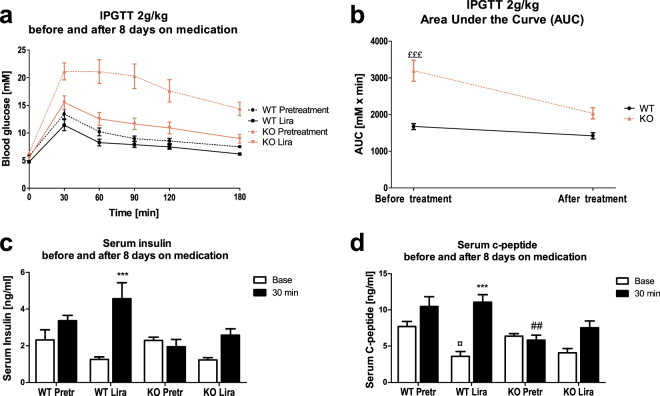
Repeated liraglutide effect on glucose intolerant 5-month-old Wfs1 KO rats. (**a**) IPGTT (2 g/kg) before and after 8-day chronic administration of 0.4 mg/kg liraglutide to 5-month-old male Wfs1 KO and control rats. (**b**) Decrease in IPGTT (2 g/kg) area under curve before and after chronic liraglutide treatment for 8 days. Glucose-stimulated increases in blood (**c**) insulin and (**d**) C-peptide levels before and 30 minutes after glucose administration. The data were compared using repeated measures ANOVA followed by Tukey’s HSD tests; ^£££^p < 0.001 compared to WT AUC during same IPGTT. ***p < 0.001 compared to baseline of the same genotype during the same IPGTT. ^##^p < 0.01 compared to WT animals in the same timepoint during the same IPGTT. ^¤^p < 0.05 compared to WT saline animals in the same timepoint during the first IPGTT. The data are presented as the mean ± SEM, n = 6–8.

In accordance with previous findings^11^, 5-month-old Wfs1 KO (Wfs1 exon 5 knock-out) rats were glucose intolerant, with peak blood glucose levels over 20 mM in response to glucose administration (Fig. 2a). Before liraglutide treatment, Wfs1 KO animals showed glucose intolerance (Fig. 2b) (F(1,12) = 28.28, p < 0.001 (genotype); F(1,12) = 63.67, p < 0.001 (treatment); F(1,12) = 26.16, p < 0.001 (genotype × treatment)); however, after 8-day repeated treatment with 0.4 mg^1^kg^−1^day^−1^ liraglutide, the difference between the genotypes disappeared (Fig. 2a); area-under-curve analysis confirmed this finding (Fig. 2b) (p > 0.05). This indicates that liraglutide improved glucose tolerance in Wfs1 KO rats (Fig. 2a,b).

Glucose-stimulated insulin secretion (Fig. 2c) was significantly weaker in KO animals 30 minutes after glucose challenge (F(1,23) = 6.07, p < 0.05 (genotype); F(1,23) = 17.38, p < 0.001 (timepoint); F(1,23) = 6.84, p < 0.05 (timepoint × genotype)). Both in wild-type (WT) and Wfs1 KO rats, the basal insulin levels were decreased and glucose-stimulated insulin secretion increased after liraglutide treatment (F(1,23) = 0.051, p = 0.82 (treatment); F(1,23) = 0.14, p = 0.71 (genotype × treatment); F(1,23) = 9.01, p < 0.01 (timepoint × treatment); F(1,25) = 0.17, p = 0.686 (timepoint × genotype × treatment)).

Similarly to glucose-stimulated insulin secretion, C-peptide levels (Fig. 2d) were significantly diminished in KO animals 30 minutes after glucose challenge (F(1,21) = 13.10, p < 0.01 (genotype); F(1,21) = 26.95, p < 0.001 (timepoint); F(1,21) = 9.1, p < 0.01 (timepoint × genotype)). Liraglutide decreased basal C-peptide levels and showed tendency toward increased C-peptide levels 30 minutes after glucose challenge, when compared to baseline (F(1,21) = 3.71, p = 0.068 (treatment); F(1,21) = 2.16, p = 0.16 (genotype × treatment); F(1,21) = 9.69, p < 0.01 (timepoint × treatment); F(1,21) = 0.008, p = 0.93 (timepoint × genotype × treatment)).

### Administration of liraglutide, from an early age inhibits the development of glucose intolerance in Wfs1 KO rats

**Figure 3:**
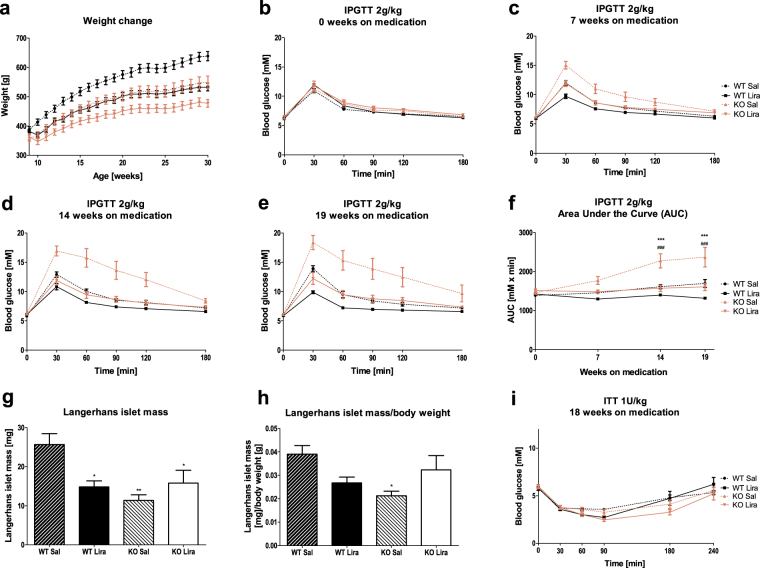
Development of glucose intolerance over 19 weeks of liraglutide treatment. (**a**) Weight change over 19 weeks of liraglutide administration. Blood glucose profile after glucose challenge during liraglutide treatment (**b**) before, (**c**) 7 weeks, (**d**) 14 weeks and (**e**) 19 weeks after the beginning of liraglutide treatment. (**f**) Area under curve analyses for IPGTT results at different timepoints. (**g**) Langerhans islet mass and (**h**) Langerhans islet mass/body weight ratio after 19 weeks of liraglutide treatment. (**i**) Insulin tolerance test with 1U/kg human insulin after 18 weeks of liraglutide treatment. The data were compared using repeated measures ANOVA or one-way ANOVA followed by Tukey’s HSD tests; *p < 0.05, **p < 0.01, ***p < 0.001 compared to (same-age) WT saline-treated animals. ^###^p < 0.001 compared to same-age Wfs1 KO liraglutide-treated animals. The data are presented as the mean ± SEM, n = 12–15 (weight and IPGTT), n = 6–8 (ITT), n = 4–6 (islet measurements).

As one-week treatment with liraglutide improved the glucose control in already glucose intolerant 5-month-old-rats, we aimed to investigate, if chronic treatment with liraglutide starting before the onset of glucose intolerance would be able to prevent or delay the development of diabetes in Wfs1 KO rats. At 2 months of age, Wfs1 KO rats’ glucose tolerance was indistinguishable from WT littermates’ (Fig. 3b). For 19 weeks of liraglutide administration, the glucose tolerance profile of Wfs1 KO rats receiving medication remained similar to that of their WT littermates, while Wfs1 KO rats in control group developed glucose intolerance (Fig. 3c–e), demonstrated by stable increase of area-under-curve (Fig. 3f) (F(1,50) = 22.598, p < 0.001 (genotype); F(1,50) = 18.27, p < 0.001 (treatment); F(1,50) = 4.047, p < 0.05 (genotype × treatment); F(3,150) = 24.26, p < 0.001 (age); F(3,150) = 11.77, p < 0.001 (age × genotype); F(3,150) = 19.82, p < 0.001 (age × treatment); F(3,150) = 5.12, p < 0.01 (age × genotype × treatment)).

Insulin sensitivity (Fig. 3i) after 18 weeks of liraglutide/saline treatment was not different between genotypes (F(1,24) = 3.36, p = 0.079) and was not affected by liraglutide treatment in either genotype (F(1,24) = 4.03, p = 0.056 (treatment); F(1,24) = 0.617, p = 0.44 (genotype × treatment)).

### Langerhans islet mass decrease is reduced in liraglutide-treated Wfs1 KO rats

Our next purpose was to assess the effect of chronic liraglutide treatment by measuring the Langerhans islet mass. Langerhans islets were visualized by staining with anti-insulin antibody. The staining effectively labeled islets in every treatment group and genotype (Supplementary Fig. 1). In agreement with a previous study^11^, Langerhans islet mass in saline-treated Wfs1 KO animals was lower than in saline-treated WT rats (Fig. 3g) (p < 0.01). Similarly to previous reports^12^, 19 week liraglutide treatment resulted in a significantly decreased islet mass in WT pancreases, compared with WT saline treated animals, but this effect was not present between Wfs1 KO treatment groups (F(1,16) = 7.58, p < 0.05 (genotype); F(1,16) = 1.72, p = 0.208 (treatment); F(1,16) = 9.93, p < 0.01 (genotype × treatment)). This opposite effect of liraglutide on Langerhans islet mass in two genotypes might be attributable to its combined action on body weight and beta cell health. Liraglutide treatment induced a decrease in body weight in both genotypes (Fig. 3a) (F(1,51) = 18.24, p < 0.001 (genotype); F(1,51) = 21.90, p < 0.001 (treatment); F(1,51) = 0.81, p = 0.37 (genotype × treatment)). Liraglutide-induced decrease in the Langerhans islet mass was paralleled by a decrease in body weight; thus, Langerhans islet mass/body weight ratio was not statistically different between liraglutide and saline treated WT rats. However, saline-treated Wfs1 KO rats had significantly lower Langerhans islet mass/body mass ratio (p < 0.05) than saline-treated WT rats (Fig. 3h). This difference was eliminated under liraglutide treatment, bringing Wfs1 KO rats’ Langerhans islet mass/body mass ratio similar to that of WT rats (F(1,16) = 2.34, p = 0.15 (genotype); F(1,16) = 0.02, p = 0.89 (treatment); F(1,16) = 8.50, p < 0.05 (genotype × treatment)).

### Liraglutide inhibits glucagon secretion induced by glucose stimulation in Wfs1 KO rats

**Figure 4:**
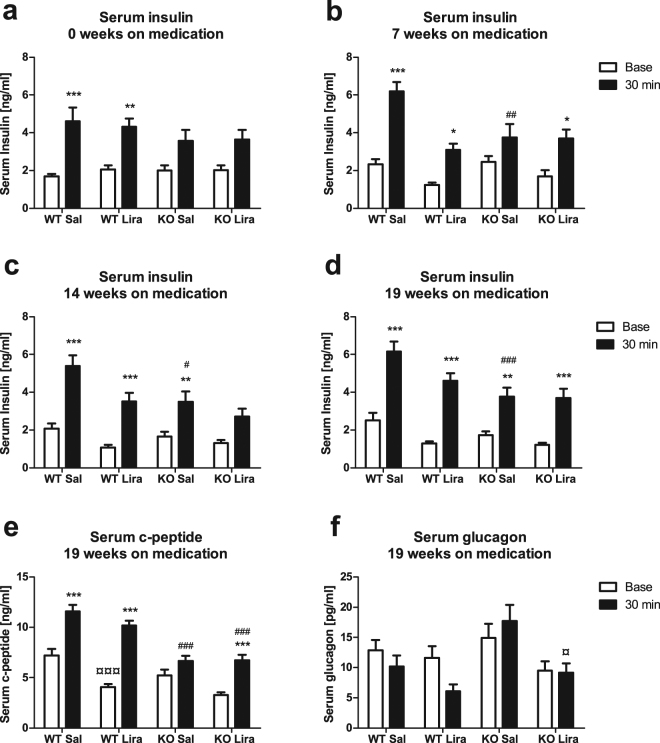
Serum insulin, C-peptide, and glucagon levels before and 30 minutes after glucose administration. (**a**–**d**) Insulin levels after various time on liraglutide treatment, (**e**) C-peptide and (**f**) glucagon levels after 19 weeks on liraglutide treatment. The data were compared using repeated measures ANOVA followed by Tukey’s HSD tests; *p < 0.05, **p < 0.01, ***p < 0.001 compared to the baseline of the same animals; ^#^p < 0.05, ^##^p < 0.01, ^###^p < 0.001 compared to WT animals of same treatment at the same timepoint; ^¤^p < 0.05, ^¤¤¤^p < 0.001 compared to saline treatment of the same GT at the same timepoint. The data are presented as the mean ± SEM, n = 12–15.

As the decrease of Langerhans islet mass appeared to be reduced in liraglutide treated Wfs1 KO rats, we next measured glucagon and insulin secretion, to assess the responsiveness of islet cells to glucose challenge. For 19 weeks, in liraglutide-treated or control rats, there were no apparent changes in insulin secretion, measured 30 minutes after glucose injection (Fig. 4a–d).

While insulin is rapidly removed from circulation by the liver, the half-life of C-peptide is at least 20 minutes^13,14^. As insulin and C-peptide are secreted in equimolar concentrations, we can presume that C-peptide concentration measured 30 minutes after glucose challenge reflects the combined secretion of first- and second-phase insulin during the whole stimulatory period. C-peptide basal levels were decreased in liraglutide-treated animals from both genotypes compared to their respective controls (F(1,51) = 12.30, p < 0.001 (treatment)). C-peptide level doubled in WT rats from both treatment groups in response to glucose. In control Wfs1 KO animals, the level of C-peptide was not significantly increased in response to glucose, but in liraglutide-treated Wfs1 KO rats, the serum C-peptide levels doubled 30 minutes after glucose administration when compared to the basal level (Fig. 4e) (F(1,51) = 37.58, p < 0.001 (genotype); F(1,51) = 2.08, p = 0.155 (treatment × genotype); F(1,51) = 202.72, p < 0.001 (timepoint); F(1,51) = 12.28, p < 0.001 (timepoint × treatment); F(1,51) = 27.38, p < 0.001 (timepoint × genotype); F(1,51) = 0.07, p = 0.79 (timepoint × treatment × genotype)).

Glucagon levels were significantly lower 30 minutes after glucose stimulation in liraglutide-treated Wfs1 KO rats than in saline-treated Wfs1 KO rats (Fig. 4f) (F(1,47) = 10.36, p < 0.01 (treatment); F(1,47) = 3.29, p = 0.076 (genotype); F(1,47) = 1.54, p = 0.22 (treatment × genotype); F(1,47) = 0.78, p = 0.383 (timepoint); F(1,47) = 0.81, p = 0.371 (timepoint × treatment); F(1,47) = 4.08, p < 0.05 (timepoint × genotype); F(1,47) = 0.32, p = 0.57 (timepoint × treatment × genotype)).

### Liraglutide treatment decreases ER stress, inflammation, and proliferation in Langerhans islets

**Figure 5:**
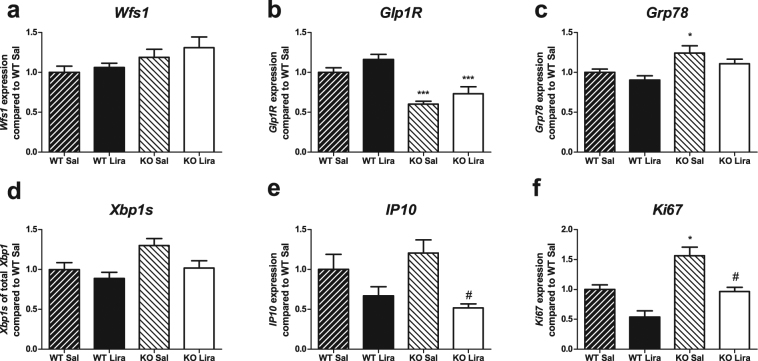
Langerhans islet gene expression analyses after 19 weeks of liraglutide treatment compared to WT saline group. Gene expression of (**a**) *Wolframin1*; (**b**) *GLP-1 receptor*; ER stress markers (**c**) *Grp78* and (**d**) *Xbp1* splicing; inflammation marker (**e**) *IP10*; proliferation marker (**f**) *Ki67*. The data were compared using factorial ANOVA followed by Tukey’s HSD tests; *p < 0.05, **p < 0.01, ***p < 0.001 compared to WT animals of the same treatment; ^#^p < 0.05, ^##^p < 0.01 compared to saline animals of the same GT. The data are presented as the mean ± SEM, n = 4–8.

To further assess the overall health of islet cells, we performed gene expression analyses of Langerhans islets, extracted from rats after 19 weeks on liraglutide/saline treatment. Gene expression revealed no significant changes in *Wfs1* expression (Fig. 5a) (F(1,25) = 5.70, p < 0.05 (genotype); F(1,25) = 0.95, p = 0.339 (treatment); F(1,25) = 0.099, p = 0.756 (genotype × treatment)).

*GLP-1* receptor (Fig. 5b) expression was decreased in Wfs1 KO animals (F(1,25) = 44.70, p < 0.001). Liraglutide had a slight increasing effect on *GLP-*1 receptor mRNA levels in both genotypes (F(1,25) = 5.64, p < 0.05 (treatment); F(1,25) = 0.056, p = 0.814 (genotype × treatment)).

The ER stress marker *Grp78* (Fig. 5c) and *Xbp1* splicing (Fig. 5d) were increased in Wfs1 KO rats compared to WT littermates (F(1,25) = 13.06, p < 0.01 and F(1,16) = 6.13, p < 0.05 respectively). Liraglutide treatment decreased ER stress (F(1,25) = 3.58, p = 0.071 and F(1,16) = 4.94, p < 0.05 respectively), resulting in similar levels in WT and liraglutide-treated KO animals (F(1,25) = 0.095, p = 0.760 (genotype × treatment) (F(1,16) = 0.95, p = 0.343 (genotype × treatment)). Reduction in ER stress was not accompanied by changes in apoptosis markers (Supplementary Fig. 2).

Inflammatory marker *IP10* (Fig. 5e) levels in Langerhans islets showed no differences between genotypes (F(1,20) = 0.03, p = 0.860). Liraglutide treatment had a significant decreasing effect on *IP10* expression, regardless of genotype (F(1,20) = 11.61, p < 0.01; F(1,20) = 1.405, p = 0.250 (genotype × treatment)).

Proliferation marker *Ki67* (Fig. 5f) expression in Wfs1 KO rats was higher than in WT littermates (F(1,18) = 17.49, p < 0.001). Liraglutide treatment decreased *Ki6*7 expression in both genotypes, bringing the expression level in KO animals similar to WT islets *Ki67* expression (F(1,18) = 20.22, p < 0.001 (treatment); F(1,18) = 0.35, p = 0.564 (genotype × treatment)).

## Discussion

Currently there is no preventive cure for WS and treatment is limited to management of symptoms^4^. In our previous studies we have shown that acute treatment with valproic acid normalized glucose intolerance in Wfs1 KO mice^15^. However, 3 months long chronic treatment with valproic acid did not improve the diabetic phenotype of Wfs1 KO mice^16^. These results highlight the importance of preclinical evaluation of long term effects of drug treatment, even if the results of short term studies seem promising. Therefore, in this study we evaluated the effects of short-term and long-term treatments with GLP-1 receptor agonist on blood glucose control in Wfs1 KO rats. The aim was to investigate whether chronic treatment with the GLP-1 receptor agonist liraglutide, starting before the onset of metabolic symptoms, would be able to prevent or delay the development of diabetes in Wfs1 KO rats. Our results indicate that chronic liraglutide treatment prevented the development of glucose intolerance in Wfs1 KO rats (Fig. 3f). Moreover, liraglutide reduced ER stress and inflammation in Wfs1 KO rats Langerhans islets (Fig. 5), which seems to result from overall better health of islet the cells and a possible protective effect of liraglutide.

Liraglutide treatment decreased basal insulin and C-peptide levels. When compared to basal levels, liraglutide-treated Wfs1 KO rats showed a twofold increase in both insulin and C-peptide levels 30 minutes after stimulation, while saline-treated Wfs1 KO rats’ C-peptide levels did not change from the basal level in response to glucose (Figs 2c,d and 4d,e). While insulin is rapidly removed from circulation by the liver, the half-life of C-peptide is at least 20 minutes^13,14^. Therefore, we can presume that C-peptide concentration measured 30 minutes after glucose challenge reflects the combined secretion of first- and second-phase insulin during the whole stimulatory period. These results indicate constant low-level insulin secretion from Wfs1 KO rats that is only slightly elevated by glucose challenge. Liraglutide treatment appears to correct that deficit by enhancing the first phase of insulin secretion. In addition to enhancement in insulin secretion, GLP-1R agonists have also been shown to reduce proinsulin:insulin ratio (elevated in subjects with type 2 diabetes), possibly as a result of reduced ER stress and therefore, improved proinsulin processing^17^. Increased proinsulin:insulin ratio has been shown in mice lacking functional Wfs1^18^. Improper proinsulin processing may also explain the lack of blood glucose lowering effect of depolarizing medication in Wfs1 deficient mice^8^. As both insulin and C-peptide ELISAs used in current study may also cross-react with proinsulin (according to our inquiry to the manufacturer), the improved glycemic control may also in part result from properly processed insulin after liraglutide treatment. These results are in accordance with those obtained from Wfs1 deficient mice, whose blood glucose levels during IPGTT are significantly lowered after treatment with the GLP-1 analogue exenatide, an effect not accompanied by increased serum insulin concentration 30 minutes after stimulation^8^. Also, administration of exenatide to Wolfram syndrome 2 (WS2, CISD2 (CDGSH Iron Sulfur Domain 2) mutation) patient lowered daily insulin dose 70% and improved glycemic control^19^. Knock-down of CISD2 by shRNA in INS-1 cells decreased insulin secretion in response to glucose or KCl by 30% and this effect was also reversed by exenatide treatment^19^. The similar response in these WS and WS2 models indicates the effectiveness of GLP-1 analogues, probably resulting from the same molecular mechanism.

The change in first-phase insulin secretion may also be at least partially responsible for the change in glucagon secretion (Fig. 4f), as intra-islet insulin, secreted from beta cells, is necessary for glucagon secretion inhibition^20–22^. Also, both liraglutide and GLP-1 have been shown to decrease glucagon levels^23,24^. Exenatide experiments with Wfs1 KO mice did not reveal any differences in glucagon secretion, but this might be due to a shorter treatment protocol or the fact that Wfs1 KO mice do not exhibit such strong progression of glucose intolerance over time^8^. Disturbances in suppression of mealtime glucagon concentration are also present in type 2 diabetes patients, whose hepatic glucose production contributes to hyperglycemia^25,26^. Changes in both insulin (as indicated by C-peptide levels) and glucagon secretion in Wfs1 KO rats are corrected by liraglutide treatment, indicating improved beta and alpha cell sensitivity and function.

Langerhans islet mass doubles in WT rats between the ages of 3 to 7 months while starting to decline in Wfs1 KO rats^11^. This decline is most likely caused by loss of beta cell mass, as in mouse model of WS, there is 50% decline in beta cell mass between 8 and 36 weeks of age, but this decline is not represented in other cell types of islet^27^. There are controversial reports regarding GLP-1 and its agonists’ effects on beta cell mass^12,28,29^. In liraglutide-treated WT rats, we saw an approximately 40% decline in Langerhans islet mass (Fig. 3g), which was very similar to the liraglutide response seen in a mouse model on hyperglycemia^12^. Interestingly, in Wfs1 KO rats, liraglutide had no decreasing effect on Langerhans islet mass, but actually seemed to prevent the decline in Wfs1 KO Langerhans islet mass, as established in our previous study^11^. Furthermore, when considering the differences in body weight, liraglutide treatment brought Wfs1 KO rats’ Langerhans islet mass closer to that of WT animals (Fig. 3h).

Even though decline in Langerhans islet mass did not occur in liraglutide-treated Wfs1 KO rats, Langerhans islet cell proliferation had significantly declined in both genotypes (Fig. 5f), indicating that the main effect in Langerhans islet mass was not caused by increased proliferation in Wfs1 KO rats. These results are in accordance with those obtained from prediabetic Goto-Kakizaki rats, whose proliferation marker Ki67 levels had also declined in response to liraglutide treatment^10^. In humans, beta cell proliferation is very low after the first few years of life, and treatments increasing proliferation in rodents are unlikely to cause the same in human beta cells^30^. Therefore, as liraglutide’s positive effect appears not to be caused by increased proliferation, it remains a promising treatment strategy for human WS patients.

Unlike Luo *et al*., we did not see a significant effect of liraglutide on islet apoptosis (Supplementary Fig. 2)^10^. This might be due to the fact that Luo *et al*. used very young rats, whose beta cells are actively proliferating and a shorter treatment protocol^10,31^. Also, reduction in beta cell mass in Wfs1 KO rat develops very slowly over time, as fasting hyperglycemia is apparent around 13 months of age, and even then, most likely there is some portion of beta cells left^11^. Moreover, we are analyzing whole islets, making it more difficult to detect differences only apparent in beta cells. In healthy rats, 20–35% islet cells are not beta cells and this ratio is even higher in aging Wfs1 KO rats^32^, who lose beta cell mass. Therefore, it is possible that the difference in rate of apoptosis is so low, that we are not able to detect it. Although there was no significant effect on apoptosis, both ER stress (Fig. 5c,d) and inflammation (Fig. 5e) were reduced by liraglutide treatment. Inflammation has long been recognized as a contributing factor in the development of diabetes^33,34^, and both GLP-1 agonists and DPP-4 inhibitors have been shown to suppress inflammation in pancreatic islets and adipose tissue^35–37^. Taken together, these results indicate better overall health of islet cells.

Diabetes mellitus is one of the first symptoms of Wolfram syndrome and is also present in a WS rat model. In this study, we demonstrated that one-week treatment with liraglutide improved glucose control in 5-month-old Wfs1 KO rats, who had already developed glucose intolerance by that age. Moreover, we have demonstrated that 19-week long treatment with Liraglutide, starting at young age before manifestation of metabolic symptoms, prevents or delays the development of diabetic phenotype in Wfs1 KO rats. As WS comprises various symptoms, starting with diabetes and eventually leading to neurodegeneration, a lifelong experiment with GLP-1 receptor agonist should follow to assess its possible positive preventive effects on other complications accompanying WS.

## Materials and Methods

### Animals

Generation and phenotype of a Wfs1 mutant (*Wfs1* exon 5 knock-out) rat has been described previously^11^. Breeding and genotyping were performed at the Laboratory Animal Centre, University of Tartu. For this study, 2- to 7-month-old and 5-month-old male homozygous Wfs1-deficient and wild-type (WT) littermate control rats were used. The animals were housed in cages in groups of 2–4 animals per cage under a 12 h light/dark cycle (lights on at 7 a.m.). Rats had unlimited access to food and water except during testing. Sniff universal mouse and rat maintenance diet (Sniff #V1534) and reverse osmosis-purified water were used. Experiments were performed between 9 a.m. and 5 p.m. Tissue collection was performed as described previously^11^. Permission for this study was given by the Estonian National Board of Animal Experiments (No 103, 22nd of May 2017) in accordance with the European Communities Directive of September 2010 (2010/63/EU).

### Intraperitoneal glucose tolerance tests (IPGTT)

Animals were deprived of food for 3 h before and during the experiment; water was available throughout the experiment. D-Glucose (Sigma-Aldrich) was dissolved in 0.9% saline solution (20% w/vol) and administered intraperitoneally at a dose of 2 g/kg of body weight. Blood glucose levels were measured at the indicated time points from the tail vein using a handheld glucometer (Accu-Check Go, Roche, Germany). Blood samples were drawn from the tail vein immediately before and 30 min after glucose administration for further analyses.

### Preliminary study: 8-day repeated liraglutide treatment

In this experiment, 5-month-old rats were used. After the first IPGTT test, the rats received 0.4 mg/kg liraglutide (Novo Nordisk, Denmark) subcutaneously for 8 days. Injections of 1 ml/kg volume were administered once a day between 8 and 11 a.m. The second IPGTT was performed 4.5 hours after the last liraglutide injection.

### Chronic liraglutide treatment

The rats were 2 months old at the beginning of the experiment. After the first IPGTT test, the rats were randomly allocated into the liraglutide or control group. The liraglutide group animals received 0.4 mg/kg liraglutide (Novo Nordisk, Denmark) and the control group animals a 0.9% saline solution (vehicle) subcutaneously. Injections of 1 ml/kg volume were made once a day between 8 and 11 a.m. (or immediately after a glucose/insulin tolerance test). Rats were weighed once a week. Glucose tolerance and insulin tolerance tests were performed 24 hours after the previous liraglutide/saline injection. After the last IPGTT, rats were equally allocated for subsequent histological/qPCR analysis according to their IPGTT results.

### Insulin, C-peptide, and glucagon measurements

For serum separation, blood was allowed to clot, centrifuged for 15 min at 2000 × g, and stored in −80 °C until further analysis. Serum insulin levels were measured using an ultra-sensitive rat insulin ELISA kit (CrystalChem cat# 90060), C-peptide levels were measured using rat C-peptide ELISA (CrystalChem cat# 90055), and glucagon levels were measured using rat glucagon ELISA (CrystalChem cat# 81519), according to the manufacturer’s instructions.

### Insulin tolerance tests

An insulin tolerance test was performed one week before the last IPGTT in the same manner as the glucose tolerance tests except that human insulin (1 unit/kg s.c., Lantus Solostar) was administered s.c. instead of glucose.

### Determination of Langerhans islet mass

Langerhans islet mass was estimated as has been described previously^38^. In brief, rats were anaesthetized with an i.p. injection of ketamine (100 mg/kg) and dexmedetomidine (250 µg/kg) and were then perfused transcardially with 4% paraformaldehyde (PFA, Sigma-Aldrich) in 0.1 M phosphate buffer (PB, pH 7.4). Their pancreases were dissected, and excess fat removed. The weight of each pancreas was recorded, and the tissue was processed for histological analysis as described previously^11^. 40-μm-thick sections were cut at intervals of 400 μm. Obtained 20–30 slices per pancreas were analysed.

Sections were washed with PBS for 3 × 5 min, permeabilized with 0.2% Triton X-100 (Naxo, Estonia)/PBS solution for 30 min, incubated in 0.5% H_2_O_2_/PBS for 1 h, and blocked in 5% donkey serum/1% bovine serum albumin (BSA, Sigma-Aldrich)/PBS for 1 h. Primary and secondary antibodies were diluted in 0.1% Tween-20/1% BSA/PBS. Sections were incubated with mouse anti-insulin antibody (1:800, Cell Signaling Technology Cat# 8138 S RRID:AB_10949314) for 1 h and washed with PBS for 3 × 10 min, followed by incubation with donkey anti-mouse peroxidase conjugated antibody (1:2000, Rockland Cat# 610-703-002 RRID:AB_219700) for 30 min and washed with PBS 3 × 5 min. Sections were incubated in 0.025% diaminobenzidine (Sigma Aldrich)/0.005% H_2_O_2_/0.05% CoCl_2_ (Sigma Aldrich)/PBS for 15 min and washed with water. Dried sections were mounted using PeRtEx (HistoLab) and covered with 0.17 mm coverslip (Deltalab). Images were taken using a Leica SCN 400 slide scanner at 20x magnification. The images obtained were analysed using the ImageJ software. Langerhans islet mass for each animal was estimated by dividing the total islet area by the total pancreas area, and the obtained relative islet area was multiplied by the weight of the pancreas to estimate Langerhans islet mass.

### Isolation of islets of Langerhans

Islets of Langerhans were isolated as has been described previously^39^. In brief, 0.9 mg/ml collagenase (Sigma-Aldrich, #C7657) solution was injected into the common bile duct of euthanized animals; inflated pancreases were collected and tissues enzymatically dispersed. Most of exocrine tissue was removed by gradient separation in Histopaque solution (Sigma-Aldrich). Islets of Langerhans were collected by hand from remaining exocrine tissue under a stereo microscope.

### RNA isolation, cDNA synthesis, and gene expression analyses

RNA from islets of Langerhans was isolated using Direct-zol RNA MiniPrep (Zymo Research), according to the manufacturer’s protocol. First-strand cDNA was synthesized using random hexamers and SuperScript™ III Reverse Transcriptase (Invitrogen, USA). qPCR using TaqMan Gene Expression Assays, and Taqman Gene Expression Mastermix (Thermo Fisher Scientific) was used to analyse *Wfs1* (Rn00582735_m1, binds exon 1 and 2 boundary, therefore also recognizes mutant *Wfs1* lacking exon 5), *Glp1R* (Glucagon-like peptide-1 receptor; Rn00562406_m1), *Grp78* (78 kDa glucose-regulated protein; Rn00565250_m1), *Atf4* (Activating transcription factor 4; Rn00824644_g1), *Bax* (Bcl-2-associated X protein; Rn01480161_g1), *Caspase1* (Rn01515235_m1), *Caspase3* (Rn00563902_m1), *Bcl2* (B-cell lymphoma 2; Rn99999125_m1), *IP10* (Interferon gamma-induced protein 10; Rn01413889_g1) and *Ki67* (Antigen KI-67; Rn01451446_m1) expression. Relative quantification was performed using the 2^−∆Ct^ method, with *Hprt1* (Hypoxanthine-guanine phosphoribosyltransferase; Rn01527840_m1) as an internal control. *Xbp1* (X-box binding protein 1) splicing was analysed using rat *Xbp1*-specific PCR as has been described previously^40^. Integrated density levels were measured using ImageJ software. All gene expression results were normalized to their expression in WT saline-treated rats.

### Data analysis

The data are presented as the mean ± SEM and were compared using factorial or repeated measures ANOVA (after Shapiro-Wilks normality test) followed by Tukey post hoc tests. The data were analysed using version 8 of the Statistica software (Statistica, USA). p < 0.05 was considered statistically significant. In some experiments, the sample size is smaller because of tissue limitations preventing a measurement from being performed, not because of the removal of data from the analysis.

## Electronic supplementary material

Supplementary Material
