# Supplementary material for: Preventive treatment with liraglutide protects against development of glucose intolerance in a rat model of Wolfram syndrome
Source: Sci Rep. 2018 Jul 5;8:10183. doi: 10.1038/s41598-018-28314-z (PMC6033861; doi:10.1038/s41598-018-28314-z)
Supplement: Supplementary file 1 — Supplementary Material [file 41598_2018_28314_MOESM1_ESM.pdf]

Supplementary Material:

Title: Preventive treatment with liraglutide protects against development of glucose intolerance in a rat model of Wolfram syndrome

Authors: Maarja Toots, Kadri Seppa, Toomas Jagomäe, Tuuliki Koppel, Maia Pallase, Indrek Heinla, Anton Terasmaa, Mario Plaas, Eero Vasar

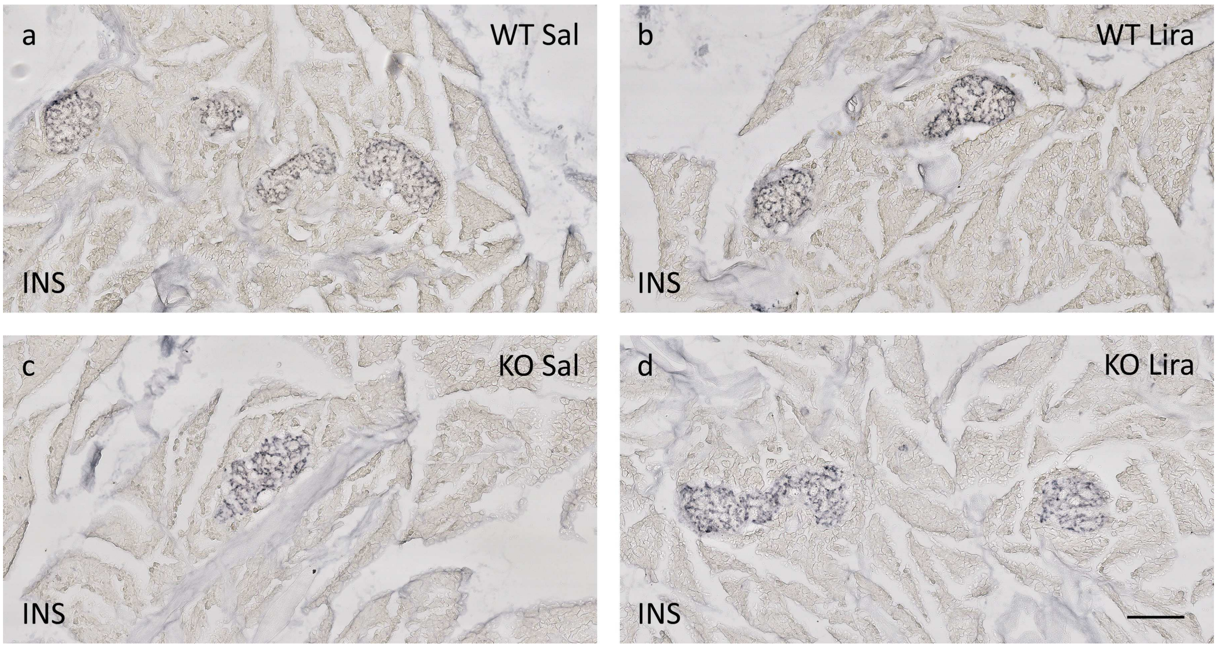

Supplementary Figure 1. Langerhans islet visualization by staining with anti-insulin antibody after 19 weeks of liraglutide/saline treatment. Staining with anti-insulin antibody effectively labeled islets in every treatment group and genotype **(a)** WT Saline, **(b)** WT Liraglutide, **(c)** KO Saline, **(d)** KO Liraglutide. INS, insulin. Scale bar 250 µm.

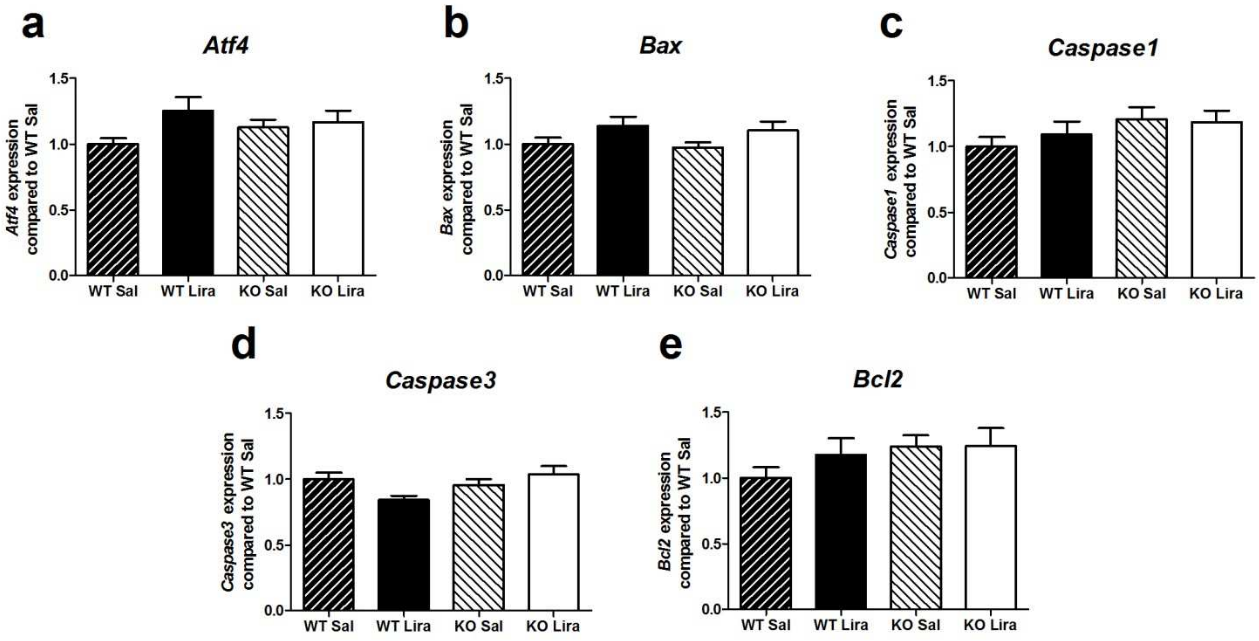

Supplementary Figure 2. Langerhans islet apoptosis marker gene expression analyses after 19 weeks of liraglutide treatment compared to WT saline group. Gene expression of apoptosis markers **(a)** *Atf4*, **(b)** *Bax*, **(c)** *Caspase 1*, **(d)** *Caspase 3,* and **(e)** *Bcl2*. The data were compared using factorial ANOVA followed by Tukey's HSD tests. The data are presented as the mean ± SEM, n=6-8
